# Intergenerational Financial Support and Planned Retirement Timing: An Overlooked Dimension of Caregiving

**DOI:** 10.1093/geroni/igaf122.590

**Published:** 2025-12-31

**Authors:** Chin-Yi Su

**Affiliations:** Boston College, Chestnut Hill, Massachusetts, United States

## Abstract

Researchers increasingly recognize that family caregiving responsibilities influence planned retirement decisions, yet most studies focus on hands-on caregiving, overlooking the impact of intergenerational financial support. This study examines how financial support to aging parents and children/grandchildren affects planned retirement timing among middle-aged and older workers. Using 2018 Health and Retirement Study (HRS) data, we analyzed a sample of 2,557 full-time and part-time workers aged 55-61. Ordered logistic regression was employed to assess the relationship between financial support to aging parents, financial support to children/grandchildren, and planned retirement age. Findings indicate that supporting aging parents significantly increases the likelihood of planning for early retirement, whereas financial support for children and grandchildren may delay retirement, though this effect is only marginally significant. Additionally, women, Black, and Hispanic older workers, as well as those with a working pension, are more likely to plan for earlier retirement. These findings highlight the subjectivity of retirement timing and the often-overlooked role of financial caregiving. While economic responsibility for younger generations may extend workforce participation, financial support for aging parents appears to accelerate retirement planning. This research underscores financial caregiving as a critical yet under examined factor shaping retirement decisions, particularly for the sandwiched generation. Future studies should further investigate the mechanisms linking financial caregiving and retirement behavior, considering how financial strain varies across caregiving roles and demographic groups.

